# Some classes of singular integral equations of convolution type in the class of exponentially increasing functions

**DOI:** 10.1186/s13660-017-1580-z

**Published:** 2017-12-16

**Authors:** Pingrun Li

**Affiliations:** School of Mathematical Sciences, Qufu Normal University, Qufu, 273165 China

**Keywords:** 45E05, 45E10, 30E25, singular integral equations, Riemann boundary value problems, dual type, convolution kernel, the class of exponentially increasing functions

## Abstract

In this article, we study some classes of singular integral equations of convolution type with Cauchy kernels in the class of exponentially increasing functions. Such equations are transformed into Riemann boundary value problems on either a straight line or two parallel straight lines by Fourier transformation. We propose one method different from the classical one for the study of such problems and obtain the general solutions and the conditions of solvability. Thus, the result in this paper improves the theory of integral equations and the classical boundary value problems for analytic functions.

## Introduction

It is well known that singular integral equations (SIEs) and integral equations of convolution type are two basic kinds of equations in the theory of integral equations. There have been many papers studying singular integral equations and a relatively complete theoretical system is almost formed (see, *e.g.*, [1–6]). These equations play important roles in other subjects and practical applications, such as engineering mechanics, physics, fracture mechanics, and elastic mechanics. For operators containing both the Cauchy principal value integral and convolution, Karapetiants-Samko [7] studied the conditions of their Noethericity in the more general case. In recent decades, many mathematicians studied some SIEs of convolution type. Litvinchuk [8] studied a class of Wiener-Hopf type integral equations with convolution and Cauchy kernel and proved the solvability of the equation. Giang-Tuan [9] studied the Noether theory of convolution type SIEs with constant coefficients. Nakazi-Yamamoto [10] proposed a class of convolution SIEs with discontinuous coefficients and transformed the equations into a Riemann boundary value problem (RBVP) by Fourier transform, and given the general solutions of the equation. Later on, Li [11] discussed the SIEs with convolution kernels and periodicity, which can be transformed into a discrete jump problems by discrete Fourier transformation, and the solvable conditions and the explicit expressions of general solutions were obtained.

The purpose of this article is to extend the theory to some classes of singular integral equations of convolution type with Cauchy kernels in the class of exponentially increasing functions. Such equations can be transformed to RBVPs with either an unknown function on a straight line or two unknown functions on two parallel straight lines by Fourier transformation. We prove the existence of the solution for the equations; moreover, the general solutions and the conditions of solvability are obtained under some conditions. Therefore, the result in this paper further generalizes the results of [7–11].

The Fourier transforms used in this paper are understood to be performed in $L^{2}(\mathbb{R})$ and the functions involved certainly belong to this space.

## Definitions and lemmas

### Definition 2.1

A function $F(x)$ belongs to class $\{\{0\}\}$, if the following two conditions are fulfilled:

(1) $F(x)\in\widehat{H}$, that is, it satisfies the Hölder condition on the whole real domain $\mathbb{R}$, including ∞, *i.e.* ±∞;

(2) $F(x)\in L^{2}(\mathbb{R})$, that is, $L^{2}(\mathbb{R})=\{F(x)\mid\int ^{+\infty}_{-\infty}| F(x)|^{2}\,dx<+\infty\}$.

### Definition 2.2

A function $f(t)$ belongs to class $\{0\}$, if its Fourier transform,

$$ F(x) =V\bigl[f(t)\bigr] =\frac{1}{\sqrt{2\pi}} \int^{+\infty}_{-\infty }f(t)e^{ixt}\,dt,\quad x\in \mathbb{R}, $$

belongs to the class $\{\{0\}\}$.

### Definition 2.3

The inverse transform $V^{-1}$ of $F(x)$ is defined by

$$ V^{-1}\bigl[F(x)\bigr] =\frac{1}{\sqrt{2\pi}} \int^{+\infty}_{-\infty }F(x)e^{-ixt}\,dx,\quad t\in\mathbb{R} $$

and we denote it as $V^{-1}[F(x)] = f(t)$.

### Definition 2.4

If there exists a real constant *τ* such that $f(t)e^{-\tau t}\in\{0\}$, we say that $f(t)$ belongs to the class of exponentially increasing functions, and we denote it as $f(t)\in\{\tau\} $, where *τ* is called the order of the exponential increase.

Denote

$$ f^{+}(t)= \textstyle\begin{cases} f(t), & t\geq0,\\ 0,& t< 0;\qquad \end{cases}\displaystyle f^{-}(t)= \textstyle\begin{cases} -f(t),& t< 0,\\ 0, & t\geq0, \end{cases} $$

obviously, $f(t)=f^{+}(t)-f^{-}(t)$.

If $f^{+}(t)\in\{\tau\}$, $f^{-}(t)\in\{\sigma\}$, then we call $f(t)\in \{\tau,\sigma\}$, where *τ* and *σ* are real constants.

### Definition 2.5

For two functions $f(x)$ and $g(x)$, their convolution is

2.1 $$ \frac{1}{\sqrt{2\pi}} \int^{+\infty}_{-\infty}f(x-s)g(s)\,ds, $$

we denote it as $f*g(x)$, where the integral (2.1) exists.

We also introduce the operator *T* of the Cauchy principal value integral as

$$ Tf(x)=\frac{1}{\pi i} \int^{+\infty}_{-\infty}\frac{f(t)}{t-x}\,dt,\quad x\in \mathbb{R}. $$

We have the following lemmas.

### Lemma 2.1

([4])

*If* $f(t)\in\{0\}$, $F(0)=0$, *then* $V(Tf(t))=-F(x)\operatorname{sgn}x$, *and* $Tf(t)\in\{0\}$.

### Lemma 2.2

*If* $f_{j}(t)\in\{p_{j},q_{j}\}$ ($j=1,2,\ldots,n$), *then*

$$\sum_{j=1}^{n}f_{j}(t)\in\Bigl\{ \max_{1\leq j\leq n}(p_{j}),\min_{1\leq j\leq n}(q_{j}) \Bigr\} . $$

### Proof

Without loss of generality, we assume that $p_{1}\geq p_{j}$ ($2\leq j\leq n$). Since

$$f_{j}^{+}(t)\in\{p_{j}\}\quad (1\leq j\leq n), \qquad e^{-p_{1}t}f_{j}^{+}(t) =e^{-(p_{1}-p_{j})t} e^{-p_{j}t}f_{j}^{+}(t), $$

and $e^{-p_{j}t}f_{j}^{+}(t)\in\{0\}$, we obtain $\sum^{n}_{j=1}f_{j}^{+}(t)\in\{p_{1}\}$, that is,

$$\sum^{n}_{j=1}f_{j}^{+}(t) \in\Bigl\{ \max_{1\leq j\leq n}(p_{j})\Bigr\} . $$

Similarly,

$$\sum^{n}_{j=1}f_{j}^{-}(t) \in\Bigl\{ \min_{1\leq j\leq n}(q_{j})\Bigr\} . $$

Lemmas 2.3-2.4 are obvious facts and we omit their proofs here. □

### Lemma 2.3

*Let* $f(t)\in\{p\}$, $f(t)\in\{q\}$, *then* $f(t)\in\{ \min (p,q), \max (p,q)\}$.

### Lemma 2.4

*Let* $h(t)\in\{\sigma,\tau\}$, $f(t)\in\{p,q\}$. *If* $p\leq\tau$, $q\geq\sigma$, *then* $h*f(t)$ *exists*, *and*

$$h*f(t)\in\bigl\{ \max (p,\sigma),\min (\tau,q)\bigr\} . $$

We introduce the following two lemmas (see [4]).

### Lemma 2.5

*If* $f(t)$, $g(t)\in\{0\}$, *then* $f*g(t)\in\{0\}$, *and* $V[f*g(t)]=F(x)G(x)$, *where* $F(x)=Vf(t)$, $G(x)=Vg(t)$.

### Lemma 2.6

*Let* $F(x)=Vf(t)$, *if* $f(t)\in\{p,q\}$, $F(ip)=F(iq)=0$, *then* $Tf(t)\in\{p,q\}$.

## Presentation of the problem

In this section we consider the following several classes of SIEs in the class of exponentially increasing functions, and we shall transform these equations into the generalized RBVPs.

(1) SIEs of dual type:

$$ \textstyle\begin{cases} a_{1}f(t)+b_{1}Tf(t)+ h_{1}*f(t)=g(t),& t\in\mathbb{R}^{+};\\ a_{2}f(t)+b_{2}Tf(t)+ h_{2}*f(t)=g(t),& t\in\mathbb{R}^{-}. \end{cases} $$

(2) Wiener-Hopf type: $af^{+}(t)+b Tf^{+}(t)+h*f^{+}(t)=g(t)$, $t\in \mathbb{R}^{+}$.

(3) One convolution kernel: $af(t)+b Tf(t)+h*f(t)=g(t)$, $t\in\mathbb{R}$.

(4) Two convolution kernels: $af(t)+ b Tf(t)+h_{1}*f^{+}(t)+h_{2}*f^{-}(t)=g(t)$, $t\in\mathbb{R}$, where *a*, *b* ($b\neq0$), $a_{j}$, $b_{j}$ are constants and $b_{j}$ are not equal to zero simultaneously. In the literature [2, 7], equations (1)-(4) were discussed in class $\{0\}$ and the general solutions and the conditions of solvability were obtained. In this paper we extend the results of [2, 7] to the class of exponentially increasing functions. Without loss of generality, we mainly study the SIEs of dual type. The method mentioned in this paper may also be applied to solving the other classes of equations.

Consider the following SIEs of dual type:

3.1 $$ \textstyle\begin{cases} a_{1}f(t)+b_{1}Tf(t)+h_{1}*f(t)=g(t),& t\in\mathbb{R}^{+};\\ a_{2}f(t)+b_{2}Tf(t)+h_{2}*f(t)=g(t),& t\in\mathbb{R}^{-}, \end{cases} $$

where $a_{j}$, $b_{j}$ are as the above, and $h_{j}(t)\in\{\sigma_{j},\tau _{j}\}$ ($j=1,2$). The known function $g(t)$ belongs to the following certain function class, and the unknown function $f(t)\in\{p,q\}$. Let $\varphi\in\{0\}$ be an undetermined function and define

$$ \varphi^{-}(t)=\frac{1}{2}(\operatorname{sgn}t-1)\varphi(t), \qquad \varphi^{+}(t)=\frac{1}{2}(\operatorname{sgn}t+1)\varphi(t), $$

obviously,

$$\varphi^{\pm}(t) \in\{0\}, \qquad \varphi^{-}(t) |_{\mathbb{R}^{+}}=0,\qquad \varphi^{+}(t)|_{\mathbb{R}^{-}}=0. $$

Extending *t* in (3.1) to $t\in\mathbb{R}$, we rewrite (3.1) as

3.2 $$ \textstyle\begin{cases} a_{1}f(t)+b_{1}Tf(t)+h_{1}*f(t)=g^{+}(t)-\varphi^{-}(t);\\ a_{2}f(t)+b_{2}Tf(t)+h_{2}*f(t)=-g^{-}(t)+\varphi^{+}(t), \end{cases}\displaystyle t\in\mathbb{R}. $$

In order to guarantee that $h_{j}*f(t)$ ($j=1,2$) exist, we apply Lemmas 2.2-2.4 and obtain

$$p=\min \{\tau_{1},\tau_{2}\}, \qquad q=\max \{\sigma _{1},\sigma_{2}\}, $$

hence

$$g\in\bigl\{ \max \{p,\sigma_{1}\}, \min \{q,\tau_{2}\}\bigr\} , \qquad \varphi\in\bigl\{ \max \{p,\sigma_{2}\},\min \{q, \tau_{1}\}\bigr\} . $$

Since the relationship of the size among $\sigma_{1}$, $\sigma_{2}$, $\tau_{1}$ and $\tau_{2}$ has 24 kinds of permutations, each permutation shall determine a different class of exponentially increasing function, and then cause different boundary value problems (BVPs). As a whole, after taking the Fourier transform for (3.2), we can obtain the following four cases.

Case 1: Equation (3.2) (or (3.1)) is transformed into the RBVP on one straight line.

Case 2: Equation (3.2) is transformed into the RBVP with two unknown functions on two parallel straight lines.

Case 3: Equation (3.2) is transformed into one-sided BVP.

Case 4: In class $\{p, q\}$, (3.2) is not solvable.

But in Case 3 it is possible that a solution of (3.1) exists under some additional conditions. By using the method of analytic continuation, this case can be transformed into Case 2, therefore, in this paper we only discuss Case 1 and Case 2.

For Case 1, without loss of generality, we only study equation (3.2) under the following condition:

$$\mbox{(a)}\quad\tau_{2}>\sigma_{2}=\tau_{1}> \sigma_{1}. $$

It follows from condition (a) that *f*, $\varphi^{\pm}$, *g*, and *Tf* belong to $\{\tau_{1}\}$. In (3.2), each term is multiplied by $e^{-t\tau_{1}}$, then all terms in the obtained equations belong to $\{ 0\}$. Taking the Fourier transform on the above obtained equations, respectively, we obtain

3.3 $$ \textstyle\begin{cases} \frac{1}{C_{1}(\xi)}F(\xi)=G^{+}(\xi)+\Phi^{-}(\xi);\\ C_{2}(\xi)F(\xi)=G^{-}(\xi)+\Phi^{+}(\xi), \end{cases} $$

where

$$\begin{aligned} & \Phi^{\pm}(\xi) = V\bigl[\varphi^{\pm}(t)\bigr],\qquad G^{\pm}(\xi)=V\bigl[g^{\pm}(t)\bigr],\qquad F(\xi)=V\bigl[f(t) \bigr], \qquad H_{j}(\xi)=V\bigl[h_{j}(t)\bigr], \\ &C_{1}(\xi)=\frac{1}{a_{1}-b_{1}\delta(\xi)+H_{1}(\xi)}, \qquad C_{2}(\xi )=a_{2}-b_{2}\delta(\xi)+H_{2}(\xi), \\ &\delta(\xi) = \textstyle\begin{cases} 1,& \operatorname{Re}\xi>0;\\ -1,& \operatorname{Re}\xi< 0, \end{cases}\displaystyle \end{aligned}$$

and $\delta(\xi)$ is obtained by taking the Fourier transform to $Tf(t)$ and applying Lemma 2.1.

By eliminating $F(\xi)$ in (3.3), we obtain the following RBVP on $\operatorname{Im} \xi=\tau_{1}$:

3.4 $$ \Phi^{+}(\xi)=C(\xi)\Phi^{-}(\xi)+D(\xi), \quad\xi=x+i\tau_{1}, x\in\mathbb{R}, $$

in which we have put

$$ C(\xi)= C_{1}(\xi)C_{2}(\xi),\qquad D(\xi)= C( \xi)G^{+}(\xi)-G^{-}(\xi). $$

For Case 2, we only solve equation (3.2) under the following condition:

$$\mbox{(b)}\quad \tau_{2}\geq\tau_{1}>\sigma_{2}\geq \sigma_{1}. $$

In this case, in order to solve (3.1), we may rewrite it as

3.5 $$\begin{aligned} &a_{1}f^{+}(t)+b_{1}Tf^{+}(t)+ h_{1}*f^{+}(t)-g^{+}(t) = a_{1}f^{-}(t)+b_{1}Tf^{-}(t)+h_{1}*f^{-}(t)- \varphi^{-}(t), \end{aligned}$$

3.6 $$\begin{aligned} & a_{2}f^{+}(t)+b_{2}Tf^{+}(t)+ h_{2}*f^{+}(t)- \varphi^{+}(t) = a_{2}f^{-}(t)+b_{2}Tf^{-}(t)+h_{2}*f^{-}(t)-g^{-}(t). \end{aligned}$$

From condition (b) we know that $f\in\{\tau_{1},\sigma_{2}\}$, $g\in\{ \tau_{1},\sigma_{2}\}$, $\varphi^{+}\in\{\tau_{1}\}$, and $\varphi ^{-}\in\{\sigma_{2}\}$. Hence, in (3.5) and (3.6), each term of the left-hand sides belongs to $\{\tau_{1}\} $,while each term of the right-hand sides belongs to $\{\sigma_{2}\}$. Let

3.7 $$\begin{aligned} & a_{1}f^{+}(t)+b_{1}Tf^{+}(t)+ h_{1}*f^{+}(t)-g^{+}(t) \\ &\quad =a_{1}f^{-}(t)+b_{1}Tf^{-}(t)+h_{1}*f^{-}(t)- \varphi^{-}(t)=e_{1}(t), \end{aligned}$$

3.8 $$\begin{aligned} & a_{2}f^{+}(t)+b_{2}Tf^{+}(t)+h_{2}*f^{+}(t)- \varphi^{+}(t) \\ &\quad =a_{2}f^{-}(t)+b_{2}Tf^{-}(t)+h_{2}*f^{-}(t)-g^{-}(t)=e_{2}(t). \end{aligned}$$

By Lemmas 2.2-2.4, we find that $e_{j}(t)$ ($j=1,2$) belong to $\{\tau _{1},\sigma_{2}\}$. Hence, we multiply each term of (3.7) and (3.8) by $e^{-t\tau_{1}}$, $e^{-t\sigma_{2}}$, respectively. Then by taking the Fourier transform on the above obtained equations, we get

3.9 $$ \textstyle\begin{cases} \frac{1}{C_{1}(\xi)}F^{+}(\xi)=G^{+}(\xi)+E_{1}(\xi);\\ C_{2}(\xi)F^{+}(\xi)=\Phi^{+}(\xi)+E_{2}(\xi), \end{cases}\displaystyle \xi=x+i\tau_{1}, x\in\mathbb{R}, $$

and

3.10 $$ \textstyle\begin{cases} \frac{1}{C_{1}(\xi)}F^{-}(\xi)=-\Phi^{-}(\xi)+E_{1}(\xi);\\ C_{2}(\xi)F^{-}(\xi)=G^{-}(\xi)+E_{2}(\xi), \end{cases}\displaystyle \xi=x+i \sigma_{2}, x\in\mathbb{R}, $$

where

$$F^{\pm}(\xi)=V\bigl[f^{\pm}(t)\bigr],\qquad E_{j}( \xi)=V\bigl[e_{j}(t)\bigr],\quad j=1,2. $$

In this paper we only consider the case of normal type, that is,

3.11 $$ H_{j}(\xi)\neq \textstyle\begin{cases} -(a_{j}-b_{j}),&\operatorname{Re}\xi>0;\\ -(a_{j}+b_{j}),&\operatorname{Re}\xi< 0, \end{cases}\displaystyle \quad \xi=x+i\tau_{1}, \xi=x+i\sigma_{2}, $$

in view of this, we have $C_{2}(\xi)\neq0$ on $\xi=x+i\sigma_{2}$. For the exceptional cases, that is, $C_{2}(\xi)=0$ on $\xi=x+i\sigma_{2}$, a method of solution is similar to the one used in [8].

By eliminating $F^{+}(\xi)$, $F^{-}(\xi)$ in (3.9), (3.10), respectively, we obtain the following RBVP with the unknown functions $\Phi(\xi)$ and $\Psi(\xi)$ on two parallel straight lines:

3.12 $$ \textstyle\begin{cases} \Phi^{+}(\xi)=C_{1}(\xi)\Psi(\xi)+G_{1}(\xi), & \xi=x+i\tau_{1};\\ \Psi(\xi)=C_{2}(\xi)\Phi^{-}(\xi)+G_{2}(\xi), & \xi=x+i\sigma_{2}, \end{cases}\displaystyle \quad x\in\mathbb{R}, $$

where

$$\begin{aligned} & G_{1}(\xi)=C(\xi)G^{+}(\xi),\qquad G_{2}(\xi)=C^{\ast}_{1}( \xi)G^{-}(\xi), \\ & \Psi(\xi)=C_{2}(\xi)E_{1}(\xi)-C^{\ast}_{1}( \xi)E_{2}(\xi),\qquad C_{1}^{*}(\xi)=\frac {1}{C_{1}(\xi)}. \end{aligned}$$

Note that BVP (3.12) is a generalization of the classical RBVPs with one unknown function.

## Methods of solutions of (3.4) and (3.12)

### On the solutions of (3.4)

Since $b_{1}$, $b_{2}$ are not equal to zero simultaneously, thus (3.4) is the RBVP with discontinuous coefficients and nodal point $i\tau_{1}$ on $\operatorname{Im}\xi=\tau_{1}$, and it can be described as follows: we want to get a function $\Phi(z)$ such that it is analytic in $\operatorname{Im} \xi>\tau_{1}$, $\operatorname{Im} \xi<\tau_{1}$, respectively, and satisfies the boundary value condition (3.4).

According to the method used in [12], take a continuous branch of $\operatorname{In}C(\xi)$ such that it is continuous at $\xi=\infty$, *e.g.*, $\operatorname{In}C(\infty)=0$, and denote

$$\gamma_{0}=\alpha_{0}+i\beta_{0} = \frac{1}{2\pi i}\bigl\{ \operatorname{In}C(i\tau _{1}+0)-\operatorname{In}C(i \tau_{1}-0)\bigr\} . $$

Then choose an integer *κ*, the index of the problem, such that $0\leq\alpha=\alpha_{0}-\kappa<1 $. Denote $\gamma=\gamma_{0}-\kappa =\alpha+i\beta_{0}$. Without loss of generality, take two points $z_{1}$, $z_{2}$ such that $\operatorname{Im}z_{1}>\operatorname{Im}\xi$, $\operatorname{Im}z_{2}<\operatorname{Im}\xi$, and assume that $z_{1}$, $z_{2}$ are a pair of symmetry points on $\operatorname{Im}\xi=\tau_{1}$, then we have $|z_{1}-i\tau_{1}|=|z_{2}-i\tau_{1}|$, $\operatorname{arg}(z_{1}-i\tau_{1})= \operatorname{arg}(z_{2}-i\tau_{1})$.

Define the following piecewise function:

4.1 $$ X(z)= \textstyle\begin{cases}e^{\Gamma(z)}, &\operatorname{Im}z>\tau_{1};\\ (\frac{z-z_{2}}{z-z_{1}})^{-\kappa}e^{\Gamma(z)},&\operatorname{Im}z< \tau_{1}, \end{cases} $$

where

4.2 $$ \Gamma(z)=\frac{1}{2\pi i} \int_{-\infty+i\tau_{1}}^{+\infty+i\tau_{1}}\frac {\ln C_{0}(t)}{t-z}\,dt,\qquad C_{0}(z)=\biggl(\frac{t-z_{2}}{t-z_{1}}\biggr)^{-\kappa}C(z), $$

in which we have taken the definite branch of

$$\operatorname{In}C_{0}(t)=-\kappa\operatorname{In}\frac{t-z_{2}}{t-z_{1}}+\operatorname{In}C(t), $$

provided that we have chosen $\operatorname{In}\frac {t-z_{2}}{t-z_{1}}|_{t=\infty}=0$, or, which is the same, $\operatorname{In}\frac{t-z_{2}}{t-z_{1}}|_{t=\pm0}=\pm i\pi$. Since $\Phi(\xi)\in\{ \{0\}\}$ and $\Phi(\infty)=0$, we get:

when $\kappa\geq0$, the general solution of (3.4) is

4.3 $$ \Phi(z)=X(z)\chi(z)+X(z)\frac{Q_{\kappa}(z)}{(z-z_{1})^{\kappa}}, $$

where

4.4 $$ \chi(z)=\frac{1}{2\pi i} \int^{+\infty+i\tau_{1}}_{-\infty+i\tau _{1}}\frac{D(t)}{X^{+}(t)(t-z)}\,dt $$

and $Q_{\kappa}(z)=q_{0}+q_{1}z+\cdots+q_{\kappa}z^{\kappa}$ is an arbitrary polynomial of degree *κ* ($\kappa\geq0$).

When $\kappa< 0$, the solution of (3.4) is still (4.3). In this case, (3.4) is solvable if and only if the conditions

4.5 $$ \int^{+\infty+i\tau_{1}}_{-\infty+i\tau_{1}}\frac {D(t)}{X^{+}(t)(t-z_{1})^{j}}\,dt=0,\quad j=1,2, \ldots,-\kappa, $$

are satisfied, and (3.4) has the unique solution (4.3) (in the sequel we understand $Q_{\kappa}(z)\equiv0$ when $\kappa< 0$). By applying the Plemelj equation (see [10]) to (4.3), we get

4.6 $$ \textstyle\begin{cases} \Phi^{+}(t)=\frac{1}{2}D(t)+X^{+}(t)\chi(t)+X^{+}(t)\frac{Q_{\kappa }(t)}{(t-z_{1})^{\kappa}};\\ \Phi^{-}(t)=-\frac{C(t)}{2D(t)}+X^{-}(t)\chi(t)+X^{-}(t)\frac{Q_{\kappa }(t)}{(t-z_{1})^{\kappa}}, \end{cases} $$

and thereby

4.7 $$ \Phi(t)=\frac{1}{2}D(t)\biggl[1+\frac{1}{C(t)}\biggr]+ \bigl[X^{+}(t)-X^{-}(t)\bigr] \biggl[\chi (t)+ \frac{Q_{\kappa}(t)}{(t-z_{1})^{\kappa}}\biggr]. $$

Since the $X^{\pm}(t)$ are bounded and $X^{\pm}(t)\neq0$, it is easy to prove that $\Phi^{\pm}(t)$, $\Phi(t)\in\{\{0\}\}$. Putting (4.6) into (3.3), we can obtain $F(x)$, thus a solution $f(t)$ of (3.4) is given by $f(t)=V^{-1}[F(x)]$.

Above all, we have the following.

#### Theorem 4.1

*Under condition* (a), *if* $h_{j}(t)\in\{\sigma _{j},\tau_{j}\}$ ($j=1,2$), *in the normal type case*, *that is*, *for* (3.11) *to be valid*, *then* (3.1) *is solvable and has the unique solution* $f(t)=V^{-1}[F(x)]$ *in* $\{p,q\}$, *where* $F(x)$ *is given by* (3.3).

### The solutions and the solvability conditions of (3.12)

In this subsection, we shall solve (3.12). It follows from Section 3 that (3.12) is the RBVP with nodes $i\tau_{1}$, $i\sigma_{2}$ on two parallel straight lines, where $\Phi^{+}(\xi)$ is the boundary value of the analytic function $\Phi(z)$, which is analytic in $\operatorname{Im}z>\tau _{1}$ and belongs to $\{\{0\}\}$ on $\xi=x+i\tau_{1}$. $\Phi^{-}(\xi)$ is the boundary value of the analytic function $\Phi(z)$, which is analytic in $\operatorname{Im}z<\sigma_{2}$ and belongs to $\{\{0\}\}$ on $\xi =x+i\sigma_{2}$; $\Psi^{\pm}(\xi)$ are the boundary values of the analytic function $\Psi (z)$, which is analytic in $\sigma_{2}<\operatorname{Im}z<\tau_{1}$ and belongs to $\{\{0\}\}$ on $\xi=x+i\tau_{1}$, $\xi=x+i\sigma_{2}$, respectively. The functions $C_{1}(\xi)$, $G_{1}(\xi)$ and $C_{2}(\xi)$, $G_{2}(\xi)$ belong to $\{\{0\} \}$ on $\xi=x+i\tau_{1}$, $\xi=x+i\sigma_{2}$, respectively. Hence, for the functions appearing in (3.2), their one-sided limits exist when $x\rightarrow\infty$ on $\xi=x+i\tau_{1}$, $\xi=x+i\sigma_{2}$. In order to unify the notations, denote $l_{1}=\tau_{1}$, $l_{2}=\sigma _{2}$. Set

$$ \gamma_{0}^{(j)}=\alpha_{0}^{(j)}+i \beta_{0}^{(j)}=\frac{1}{2\pi i}\bigl\{ \operatorname{In}C_{j}(il_{j}+0)-\operatorname{In}C_{j}(il_{j}-0) \bigr\} , \quad j=1,2, $$

then choose integers $\kappa_{j}$ ($j=1,2$), such that $0\leq\alpha _{0}^{(j)}-\kappa_{j}<1$. Denote

$$\gamma_{j}=\gamma_{0}^{(j)}-\kappa_{j}= \alpha^{(j)}+i\beta_{0}^{(j)}, $$

we call $\kappa=\kappa_{1}+\kappa_{2}$ the index of (3.1). Set

$$\begin{aligned} & \gamma_{\infty}^{(j)}=\alpha_{\infty}^{(j)}+i \beta_{\infty}^{(j)}=\frac {1}{2\pi i}\bigl\{ \operatorname{In}C_{j}(il_{j}+ \infty)-\operatorname{In}C_{j}(il_{j}-\infty )\bigr\} , \\ &\alpha_{\infty}=\alpha_{\infty}^{(1)}+\alpha_{\infty}^{(2)}, \quad j=1,2. \end{aligned}$$

Note that $\operatorname{In} C_{j}(\xi)$ is taken to be continuous in $\operatorname{Re}\xi>0$ and $\operatorname{Re}\xi<0$, respectively, such that $0\leq\alpha _{\infty}<1$. Without loss of generality, we take the three points $z_{0}$, $z_{1}$, $z_{2}$ such that $l_{2}<\operatorname{Im}z_{0}<l_{1}$, $\operatorname{Im}z_{1}>l_{1}$, $\operatorname{Im}z_{2}< l_{2}$, and $|z_{0}-il_{2}|=|z_{2}-il_{2}|$, $|z_{0}-il_{1}|=|z_{1}-il_{1}|$.

In order to solve (3.12), we define the following piecewise functions:

4.8 $$\begin{aligned} & X_{1}(z)= \textstyle\begin{cases}e^{\Gamma_{1}(z)},&\operatorname{Im}z>l_{1},\\ (\frac{z-z_{1}}{z-z_{0}})^{-\kappa_{1}}e^{\Gamma_{1}(z)},& \operatorname{Im}z< l_{1}; \end{cases}\displaystyle \\ \\ & X_{2}(z)= \textstyle\begin{cases}(\frac{z-z_{2}}{z-z_{0}})^{-\kappa_{2}}e^{\Gamma _{2}(z)},& \operatorname{Im}z>l_{2},\\ e^{\Gamma_{2}(z)},&\operatorname{Im}z< l_{2}, \end{cases}\displaystyle \end{aligned}$$

where

$$\begin{aligned} & \Gamma_{j}(z)=\frac{1}{2\pi i} \int^{+\infty+il_{j}}_{-\infty +il_{j}}\biggl(\frac{\operatorname{ln}M_{j}(t)}{t-z}- \frac{\operatorname{ln}M_{j}(t)}{t-z_{0}}\biggr)\,dt, \\ & M_{j}(t)=\biggl(\frac{t-z_{j}}{t-z_{0}}\biggr)^{-\kappa_{j}}C_{j}(t), \qquad \operatorname{Im}z\neq l_{j},\quad j=1,2, \end{aligned}$$

here $\operatorname{ln}M_{j}(t)$ ($j=1,2$) have certain analytic branches such that $\operatorname{ln}\frac{t-z_{0}}{t-z_{j}}|_{t=\infty}=0$ ($j=1,2$). Setting $X(z)=X_{1}(z)X_{2}(z)$, we call $X(z)$ the canonical function of (3.12). Taking the boundary values for $X_{j}(z)$ in (4.8), we obtain by applying Plemelj’s formula

4.9 $$ X_{j}^{+}(t)=C_{j}(t)X_{j}^{-}(t), \quad j=1,2. $$

Putting (4.9) into (3.12), one has

4.10 $$ \textstyle\begin{cases} \Phi^{+}(\xi)=\frac{X_{1}^{+}(\xi)}{X_{1}^{-}(\xi)}\Psi(\xi)+G_{1}(\xi), & \xi\in L_{1}:\xi=x+il_{1};\\ \Psi(\xi)=\frac{X_{2}^{+}(\xi)}{X_{2}^{-}(\xi)}\Phi^{-}(\xi)+G_{2}(\xi), & \xi\in L_{2}:\xi=x+il_{2}, \end{cases}\displaystyle \quad x\in\mathbb{R}, $$

by multiplying $[X^{+}(\xi)]^{-1}$ to the first equality of (4.10) and $[\tilde{X}(\xi)]^{-1}$ to the second one, we have

4.11 $$ \textstyle\begin{cases} \frac{\Phi^{+}(\xi)}{X^{+}(\xi)}=\frac{\Psi(\xi)}{\widetilde{X}(\xi)}+\frac {G_{1}(\xi)}{X^{+}(\xi)}, &\xi\in L_{1};\\ \frac{\Psi(\xi)}{\widetilde{X}(\xi)}=\frac{\Phi^{-}(\xi)}{X^{-}(\xi )}+\frac{G_{2}(\xi)}{\widetilde{X}(\xi)}, &\xi\in L_{2}, \end{cases} $$

where $\widetilde{X}(\xi)=X^{-}_{1}(\xi)X^{+}_{2}(\xi)$. Since $X_{1}(\xi)$ and $X_{2}(\xi)$ are bounded on $L_{1}$, $L_{2}$, respectively, and $G_{1}(\xi )\in\{\{0\}\}$, we have $\frac{G_{1}(\xi)}{X^{+}(\xi)}\in\{\{0\}\}$, thus we may define the following piecewise function:

4.12 $$ \psi_{1}(z)=\frac{1}{2\pi i} \int^{+\infty+il_{1}}_{-\infty+il_{1}}\frac {(z-z_{0})G_{1}(t)}{X^{+}(t)(t-z)(t-z_{0})}\,dt,\quad \operatorname{Im}z\neq l_{1}. $$

It follows from Privalov’s theorem (see [12]) that $\psi_{1}(z)$ is analytic in $\operatorname{Im}z>l_{1}$ and $\operatorname{Im}z< l_{1}$, respectively. By applying Plemelj’s formula to $\psi_{1}(z)$ in (4.12), we have

$$\psi_{1}^{+}(\xi)-\psi_{1}^{-}(\xi)=\frac{G_{1}(\xi)}{X^{+}(\xi)},\quad \xi\in L_{1}. $$

Then the first equality of (4.11) can be transformed to

$$\frac{\Phi^{+}(\xi)}{X^{+}(\xi)}-\psi_{1}^{+}(\xi)=\frac{\Psi(\xi)}{\widetilde {X}(\xi)}- \psi_{1}^{-}(\xi), \quad\xi\in L_{1}. $$

Again we define

4.13 $$ \psi_{2}(z)=\frac{1}{2\pi i} \int^{+\infty+il_{2}}_{-\infty+il_{2}}\frac {(z-z_{0})G_{2}(t)}{\widetilde{X}(t)(t-z)(t-z_{0})}\,dt,\quad \operatorname{Im}z\neq l_{2}. $$

Similarly, the second equality of (4.11) can also be transformed to

$$\frac{\Psi(\xi)}{\widetilde{X}(\xi)}-\psi_{2}^{+}(\xi)=\frac{\Phi^{-}(\xi )}{X^{-}(\xi)}- \psi_{2}^{-}(\xi),\quad\xi\in L_{2}. $$

Thus, we need only to discuss (4.14) instead of (3.12):

4.14 $$ \textstyle\begin{cases} \frac{\Phi^{+}(\xi)}{X^{+}(\xi)}-\psi_{1}^{+}(\xi)=\frac{\Psi(\xi)}{\widetilde {X}(\xi)}-\psi_{1}^{-}(\xi);\\ \frac{\Psi(\xi)}{\widetilde{X}(\xi)}-\psi_{2}^{+}(\xi)=\frac{\Phi^{-}(\xi )}{X^{-}(\xi)}-\psi_{2}^{-}(\xi), \end{cases} $$

by subtracting $\psi_{2}^{+}(\xi)$ in the first equality of (4.14) and $\psi_{1}^{-}(\xi)$ in the second one, we have

4.14′ $$ \textstyle\begin{cases} \frac{\Phi^{+}(\xi)}{X^{+}(\xi)}-\psi_{1}^{+}(\xi)-\psi_{2}^{+}(\xi)=\frac{\Psi(\xi )}{\widetilde{X}(\xi)}-\psi_{1}^{-}(\xi)-\psi_{2}^{+}(\xi);\\ \frac{\Psi(\xi)}{\widetilde{X}(\xi)}-\psi_{2}^{+}(\xi)-\psi_{1}^{-}(\xi)=\frac {\Phi^{-}(\xi)}{X^{-}(\xi)}-\psi_{2}^{-}(\xi)-\psi_{1}^{-}(\xi). \end{cases} $$

In (4.14′), the left-hand side of the first equality is denoted by $\Phi _{1}^{+}$, while the right-hand side is denoted by $\Phi_{1}^{-}$; the left-hand side of the second equality is denoted by $\Phi_{2}^{+}$, while the right-hand side is denoted by $\Phi_{2}^{-}$. Thus we may denote

$$ \Phi_{1}(z)= \textstyle\begin{cases} \Phi_{1}^{+}(z),&\operatorname{Im}z>l_{1};\\ \Phi_{1}^{-}(z),&\operatorname{Im}z< l_{1}, \end{cases} $$

where

$$\begin{aligned} & \Phi_{1}^{+}(z)=\frac{\Phi^{+}(z)}{X^{+}(z)}-\psi_{1}^{+}(z)- \psi_{2}^{+}(z),\quad \operatorname{Im} z>l_{1}, \\ & \Phi_{1}^{-}(z)=\frac{\Psi(z)}{\widetilde{X}(z)}-\psi_{1}^{-}(z)- \Psi_{2}^{+}(z),\quad \operatorname{Im} z< l_{1}. \end{aligned}$$

Obviously $\Phi_{1}^{+}(z)=\Phi_{1}^{-}(z)$ on $\operatorname{Im}z=l_{1}$. When $\kappa >0$, $[\widetilde{X}(z)]^{-1}$ has a *κ*-order pole-point at $z=z_{0}$ in $l_{2}<\operatorname{Im}z<l_{1}$; when $\kappa< 0$, $[\widetilde {X}(z)]^{-1}$ has no singularity in $l_{2}<\operatorname{Im}z<l_{1}$, but $\widetilde{X}(z) $ has a $|\kappa|$-order pole-point at $z_{0}$. Therefore, by extended Liouville theory [13], we obtain

$$\Phi_{1}(z)=\frac{P_{\kappa}(z)}{(z-z_{0})^{\kappa}}, $$

where $P_{\kappa}(z)=c_{0}+c_{1}z+c_{2}z^{2}+\cdots+c_{\kappa}z^{\kappa}$, and $c_{j}$ ($0\leq j\leq\kappa$) are arbitrary constants. When $\kappa>-1$, $P_{\kappa}(z)$ is a polynomial with degree *κ*; when $\kappa\leq-1$, $P_{\kappa}(z)\equiv0$. Therefore, one has

$$ \textstyle\begin{cases} \frac{\Phi^{+}(z)}{X^{+}(z)}-\psi_{1}^{+}(z)-\psi_{2}^{+}(z)=\frac{P_{\kappa}(z)}{(z-z_{0})^{\kappa}}, &\operatorname{Im}z>l_{1},\\ \frac{\Psi(z)}{\widetilde{X}(z)}-\psi_{1}^{-}(z)-\Psi_{2}^{+}(z)=\frac{P_{\kappa}(z)}{(z-z_{0})^{\kappa}}, &\operatorname{Im}z< l_{1}, \end{cases} $$

that is,

$$ \textstyle\begin{cases} \Phi^{+}(z)=X^{+}(z)[\psi_{1}^{+}(z)+\psi_{2}^{+}(z)+\frac{P_{\kappa}(z)}{(z-z_{0})^{\kappa}}], &\operatorname{Im}z>l_{1},\\ \Psi(z)=\widetilde{X}(z)[\psi_{1}^{-}(z)+\psi_{2}^{+}(z)+\frac{P_{\kappa}(z)}{(z-z_{0})^{\kappa}}], &\operatorname{Im}z< l_{1}. \end{cases} $$

Similarly, we have

$$\textstyle\begin{cases} \Phi^{-}(z)=X^{-}(z)[\psi_{1}^{-}(z)+\psi_{2}^{-}(z)+\frac{P_{\kappa}(z)}{(z-z_{0})^{\kappa}}], &\operatorname{Im}z< l_{2},\\ \Psi(z)=\widetilde{X}(z)[\psi_{1}^{-}(z)+\psi_{2}^{+}(z)+\frac{P_{\kappa}(z)}{(z-z_{0})^{\kappa}}], &\operatorname{Im}z>l_{2}. \end{cases} $$

From the above discussions, we can obtain the solutions of (4.11):

4.15 $$ \textstyle\begin{cases} \Phi^{+}(z)=X^{+}(z)[\psi_{1}^{+}(z)+\psi_{2}^{+}(z)+\frac{P_{\kappa}(z)}{(z-z_{0})^{\kappa}}], &\operatorname{Im}z>l_{1},\\ \Phi^{-}(z)=X^{-}(z)[\psi_{1}^{-}(z)+\psi_{2}^{-}(z)+\frac{P_{\kappa}(z)}{(z-z_{0})^{\kappa}}], &\operatorname{Im}z< l_{2}, \\ \Psi(z)=\widetilde{X} (z)[\psi_{1}^{-}(z)+\psi_{2}^{+}(z)+\frac{P_{\kappa}(z)}{(z-z_{0})^{\kappa}}],& l_{2}< \operatorname{Im}z< l_{1}, \end{cases} $$

where $\psi_{j}^{\pm}(z)$ ($j=1,2$), defined by (4.12) and (4.13), respectively, are analytic functions in $\operatorname{Im}z>l_{j}$, $\operatorname{Im}z< l_{j}$.

Putting $\Phi^{+}(z)$ and $\Phi^{-}(z)$ into the second equation of (3.9) and the second one of (3.10), respectively, and denoting

$$ B(\xi)=\frac{E_{2}(\xi)}{C_{2}(\xi)},\qquad S(\xi)=\frac{\Phi^{+}(\xi)}{C_{2}(\xi )},\qquad W(\xi)= \frac{G^{-}(\xi)}{C_{2}(\xi)}, $$

then we obtain the following RBVP with two unknown functions $F(\xi)$, $B(\xi)$ on two parallel straight lines:

4.16 $$ \textstyle\begin{cases} F^{+}(\xi)=B(\xi)+S(\xi), &\xi\in L_{1};\\ F^{-}(\xi)=B(\xi)+W(\xi), &\xi\in L_{2}. \end{cases} $$

It is easy to see that the zero-points $z_{1}^{*}, z_{2}^{*},\ldots,z_{\mu}^{*}$ of $C_{2}(z)$, with the orders $s_{1},s_{2},\ldots,s_{\mu}$, respectively, are the pole-points of $B(z)$ in $l_{2}<\operatorname{Im}z<l_{1}$. Similar to the method for solving $\Phi^{\pm}(z)$ and $\Psi(z)$, we obtain the solutions of (4.16) as follows:

4.17 $$\begin{aligned} & F^{+}(z)=\frac{1}{2\pi i} \int_{-\infty+il_{1}}^{+\infty+il_{1}}\frac {S(t)}{t-z}\,dt-\frac{1}{2\pi i} \int_{-\infty+il_{1}}^{+\infty+il_{1}}\frac {S(t)}{t-z_{0}}\,dt+P_{\kappa}(z), \quad\operatorname{Im}z>l_{1}, \end{aligned}$$

4.18 $$\begin{aligned} & F^{-}(z)=\frac{1}{2\pi i} \int_{-\infty+il_{2}}^{+\infty+il_{2}}\frac {W(t)}{t-z}\,dt-\frac{1}{2\pi i} \int_{-\infty+il_{2}}^{+\infty+il_{2}}\frac {W(t)}{t-z_{0}}\,dt+P_{\kappa}(z), \quad\operatorname{Im}z< l_{2}, \end{aligned}$$

and

$$ B(z)=F^{+}(z)-F^{-}(z)+\Delta(z),\quad l_{2}< \operatorname{Im}z< l_{1}, $$

where

$$\Delta(z)=\frac{\tilde{P}_{s}(z)}{\prod_{j=1}^{\mu}(z-z_{j}^{*})^{s_{j}}}, \quad s=\sum_{j=1}^{\mu}s_{j}, $$

$P_{\kappa}(z)$ is defined above, and $\tilde{P}_{s}(z)$ is an arbitrary polynomial of degree *s*.

Therefore, a solution of (3.1) is

4.19 $$ f(t)=\frac{1}{\sqrt{2\pi}} \int_{-\infty+il_{1}}^{+\infty+il_{1}}F^{+}(\xi )e^{-i\xi t}\,d\xi- \frac{1}{\sqrt{2\pi}} \int_{-\infty+il_{2}}^{+\infty +il_{2}}F^{-}(\xi)e^{-i\xi t}\,d\xi, $$

where $F^{+}(\xi)$ takes the positive boundary value of (4.17), and $F^{-}(\xi)$ takes the negative boundary value of (4.18).

Next, we come to discuss the solvability conditions for equation (3.1).

(1) If $C_{1}(z)C_{2}(z)\neq0$ in $l_{2}<\operatorname{Im}z<l_{1}$, we can obtain $\Phi ^{\pm}(z)$ and $\Psi(z)$ from (4.15).

(2) When $\kappa<0$, $X_{1}^{-}(z)X_{2}^{+}(z)$ (that is, $\tilde{X}(z)$) has a singularity at $z=z_{0}$, then the following conditions are fulfilled:

4.20 $$ \int_{-\infty+il_{1}}^{+\infty+il_{1}}\frac {G_{1}(t)}{X^{+}(t)(t-z_{0})^{j}}\,dt- \int_{-\infty+il_{2}}^{+\infty+il_{2}}\frac {G_{2}(t)}{\tilde{X}(t)(t-z_{0})^{j}}\,dt=0, $$

for any $j=1,2,\ldots,-\kappa$.

(3) If $z_{1}^{**}, z_{2}^{**},\ldots,z_{\nu}^{**}$ are common zero-points of $C_{1}^{*}(z)$ and $C_{2}(z)$ with the orders $s_{1},s_{2},\ldots, s_{\nu}$ ($s_{j}\in\mathbb{N}$), respectively, in $l_{2}<\operatorname{Im}z<l_{1}$, then, in order to guarantee that (3.1) has a solution, we require

$$\Psi^{(j)}\bigl(z_{t}^{**}\bigr)=0,\quad1\leq t \leq\nu, 1\leq j\leq s_{t}. $$

In this case, we also have the following results:

when $\kappa\geq0 $, equations (4.21) with the unknown elements $c_{0},c_{1},\ldots,c_{\kappa}$ have solutions,

4.21 $$\begin{aligned} &\frac{j!}{2\pi i}\biggl[ \int_{-\infty+il_{2}}^{+\infty+il_{2}}\frac{G_{2}(\xi )}{(\xi-z_{0})\tilde{X}(\xi)(\xi-z_{t}^{*})^{j+1}}\,d\xi+ \int_{-\infty +il_{1}}^{+\infty+il_{1}}\frac{G_{1}(\xi)}{(\xi-z_{0})X^{+}(\xi)(\xi -z_{t}^{*})^{j+1}}\,d\xi\biggr] \\ &\quad =\biggl[\frac{P_{\kappa}(z)}{(z-z_{0})^{\kappa}}\biggr]_{z=z_{t}^{*}}^{(j)},\quad j=0,1,2, \ldots,s_{t}; t=1,2,\ldots,\mu, \end{aligned}$$

where $c_{0},c_{1},\ldots,c_{\kappa}$ are the coefficients of $P_{\kappa}(z)$, and (4.21) contain $s+\mu$ equations with $\kappa+1$ unknown elements.

When $\kappa<0$, the following equalities are fulfilled:

4.22 $$ \int_{-\infty+il_{2}}^{+\infty+il_{2}}\frac{G_{2}(\xi)}{(\xi-z_{0})\tilde{X}(\xi )(\xi-z_{k}^{*})^{j+1}}\,d\xi+ \int_{-\infty+il_{1}}^{+\infty+il_{1}}\frac{G_{1}(\xi )}{(\xi-z_{0})X^{+}(\xi)(\xi-z_{k}^{*})^{j+1}}\,d\xi=0 $$

for any $j=0,1,2,\ldots,s_{t}$; $t=1,2,\ldots,\mu$.

Thus, we have the following conclusion as regards the solution of equation (3.1).

#### Theorem 4.2

*Under condition* (b), *in the case of normal type*, *if* $C_{1}(z)C_{2}(z)\neq0$ *in* $l_{2}<\operatorname{Im}z<l_{1}$, *then the solvable condition of* (3.1) *is* (4.20). *If* $C_{1}^{*}(z)$, $C_{2}(z)$ *have some common zero*-*points* $z_{1}^{**},z_{2}^{**},\ldots,z_{\nu}^{**}$ *in* $l_{2}<\operatorname{Im}z<l_{1}$, *then* (4.21) *and* (4.22) *must be augmented*. *Thus*, *a solution* $f(t)$ *of* (3.1) *is given by* (4.19), *in which* $F^{+}(\xi)$, $F^{-}(\xi)$ *are obtained by* (4.17) *and* (4.18), *respectively*. *It is easy to verify that* $f(t)\in\{ p,q\}$.

Finally, we remark that the method of this paper may be applied to solving the equations mentioned above in the non-normal cases (or, the exceptional cases), that is,

$$ H_{j}(\xi)= \textstyle\begin{cases} -(a_{j}-b_{j}),&\operatorname{Re}\xi>0;\\ -(a_{j}+b_{j}),&\operatorname{Re}\xi< 0, \end{cases}\displaystyle \quad j=1,2; \xi\in L_{1}, L_{2}. $$

As for the method of solution for this case, there is no essential difference for the solving method with the normal case. We will not elaborate on that here.

## Results and discussion

In this article, some classes of SIEs of convolution type with Cauchy kernels are solved in the class of exponentially increasing functions. By Fourier transform, such equations are transformed into RBVPs on either a straight line or two parallel straight lines. The exact solutions of equation (3.1), denoted by integrals and the conditions of solvability are obtained. Here, our method is different from the ones for the classical boundary value problem, and it is novel and effective. Thus, the result in this paper generalizes the theory of classical boundary value problems and singular integral equations. Similarly, the above equations can also be solved in Clifford analysis (see [14–17]). Further discussion is omitted here.

## Conclusions

In this paper, we mainly study the singular integral equations of dual type in the class of exponentially increasing functions. This class of equations (that is, equation (3.1)) have important applications in practical problems, such as elastic mechanics, heat conduction, and electrostatics. Hence, the study of equation (3.1) is of significance not only in applications but also in the theory of resolving the equation itself. To many problems, such as piezoelectric material, voltage magnetic materials and functional gradient materials, one can often attribute the problem to finding solutions for this classes of equations. Hence, the result in this paper improves some results in Refs. [2, 4, 9–11, 18, 19], and it supplies a theoretical basis for solving the physics problems involved.
